# Microparticles derived from human erythropoietin mRNA-transfected mesenchymal stem cells inhibit epithelial-to-mesenchymal transition and ameliorate renal interstitial fibrosis

**DOI:** 10.1186/s13287-020-01932-z

**Published:** 2020-09-29

**Authors:** Mirae Lee, Seok-hyung Kim, Jong Hyun Jhee, Tae Yeon Kim, Hoon Young Choi, Hyung Jong Kim, Hyeong Cheon Park

**Affiliations:** 1 Graduate Program of Nano Science and Technology, Yonsei University, Seoul, Korea; 2 Division of Nephrology, Department of Internal Medicine, Gangnam Severance Hospital, Yonsei University College of Medicine, Seoul, Korea; 3 Division of Nephrology, Department of Internal Medicine, Hallym University Chuncheon Sacred Heart Hospital, Hallym University College of Medicine, Chuncheon, Gangwon-do Korea; 4 Severance Institute for Vascular and Metabolic Research, Yonsei University College of Medicine, Seoul, Korea; 5 Department of Internal Medicine, CHA Bundang Medical Center, CHA University, Seongnam, Gyeonggi-do Korea

**Keywords:** Microparticles, Transforming growth factor-β1, Renal fibrosis, Epithelial-to-mesenchymal transition, Erythropoietin

## Abstract

**Background:**

Renal tubulointerstitial fibrosis (TIF) plays an important role in the progression of chronic kidney disease (CKD) and its pathogenesis involves epithelial-to-mesenchymal transition (EMT) upon renal injury. Recombinant human erythropoietin (rhEPO) has been shown to display novel cytoprotective effects, in part by inhibiting transforming growth factor (TGF)-β1-induced EMT. Here, we evaluated the inhibitory effects of microparticles (MPs) derived from human EPO gene-transfected kidney mesenchymal stem cells (hEPO-KMSCs) against TGF-β1-induced EMT in Madin-Darby canine kidney (MDCK) cells and against TIF in mouse kidneys with unilateral ureteral obstruction (UUO).

**Methods:**

EMT was induced in MDCK cells by treatment with TGF-β1 (5 ng/mL) for 48 h and then inhibited by co-treatment with rhEPO (100 IU/mL), mock gene-transfected KMSC-derived MPs (MOCK-MPs), or hEPO-KMSC-derived MPs (hEPO-MPs) for a further 48 h. UUO was induced in FVB/N mice, which were then treated with rhEPO (1000 IU/kg, intraperitoneally, every other day for 1 week), MOCK-MPs, or hEPO-MPs (80 μg, intravenously). Alpha-smooth muscle actin (α-SMA), fibronectin, and E-cadherin expression were evaluated in MDCK cells and kidney tissues, and the extent of TIF in UUO kidneys was assessed by immunohistochemical staining.

**Results:**

TGF-β1 treatment significantly increased α-SMA and fibronectin expression in MDCK cells and decreased that of E-cadherin, while co-treatment with rhEPO, MOCK-MPs, or hEPO-MPs markedly attenuated these changes. In addition, rhEPO and hEPO-MP treatment effectively decreased phosphorylated Smad2 and Smad3, as well as phosphorylated p38 mitogen-activated protein kinase (MAPK) expression, suggesting that rhEPO and rhEPO-MPs can inhibit TGF-β1-induced EMT via both Smad and non-Smad pathways. rhEPO and hEPO-MP treatment also significantly attenuated the extent of renal TIF after 1 week of UUO compared to MOCK-MPs, with hEPO-MPs significantly reducing myofibroblast and F4/80+ macrophage infiltration as well as EMT marker expression in UUO renal tissues in a similar manner to rhEPO.

**Conclusions:**

Our results demonstrate that hEPO-MPs modulate TGF-β1-induced EMT in MDCK cells via the Smad2, Smad3, and p38 MAPK pathways and significantly attenuated renal TIF in UUO kidneys.

## Background

Renal tubulointerstitial fibrosis (TIF) is characterized by a complex process involving inflammatory cell infiltration, loss of renal tubule epithelial cells (TECs), epithelial-to-mesenchymal transition (EMT), myofibroblast accumulation, and excessive extracellular matrix (ECM) deposition [1]. Renal tubular EMT is thought to be an important mechanism that leads to TIF, with recent studies finding that some interstitial alpha-smooth muscle actin (α-SMA)-positive myofibroblast populations are derived from TECs via EMT [2, 3]. Transforming growth factor beta (TGF-β) is a pivotal mediator of renal TIF pathogenesis [4, 5], and detailed studies in both experimental animal and human disease models have demonstrated that TGF-β1 and its receptors are highly upregulated and stimulate EMT in renal TIF [6]. Indeed, experimental TGF-β inhibition has been shown to significantly reduce the expression of EMT markers and ameliorate TIF [7, 8]. For instance, hepatocyte growth factor and bone morphogenic protein-7 inhibit α-SMA overexpression and E-cadherin underexpression triggered by TGF-β1, thus preventing renal interstitial fibrosis in obstructed kidneys [9, 10]. As such, interventions that block or inhibit EMT could be potential therapeutic strategies for treating chronic progressive renal fibrosis.

Erythropoietin (EPO) is a 30.4-kDa glycoprotein hormone produced primarily by the kidneys that regulates red blood cell production in response to anemia [11]. In addition to its hematopoietic effects, recent studies have highlighted the cytoprotective effects of EPO in kidney tissues [12], while EPO has been shown to ameliorate cisplatin-induced and ischemia-reperfusion renal injuries without affecting hemoglobin [13] and inhibit the apoptosis of TGF-β1-treated LLC-PK1 cells in vitro [4, 14]. EPO treatment has also been found to reduce renal fibrosis in a murine model of unilateral ureteral obstruction (UUO) by inhibiting TGF-β1-induced EMT [15], suggesting that inhibition of EMT by EPO administration could be a potential therapeutic strategy to inhibit or slow CKD progression. Indeed, chronic administration of low-dose long-acting EPO was found to ameliorate tubulointerstitial damage and preserve renal function in 5/6 remnant kidney models [16]. However, prolonged infusion of low-dose EPO is technically difficult and impractical in a clinical environment. In addition, clinical application of soluble growth factor proteins such as EPO is limited by their short-half life, rapid enzymatic inactivation, and inefficient delivery to target tissues [17]. Moreover, the direct injection of high doses of growth factors to achieve an effective local concentration in target tissues may cause unexpected side effects in vivo. To overcome this challenge, recent studies have used genetically modified mesenchymal stem cells (MSCs) that secrete EPO (MSC-EPO) to deliver EPO to target tissues [18, 19]; however, their poor long-term cellular viability and potential induction of anti-EPO antibodies have limited their widespread use [20].

Microparticles (MPs) are submicron-sized vesicles released by a variety of cells, including stem cells, in response to oxidative stress or hypoxia. Stem cell-derived MPs can mimic the biological activities of the original stem cells and interact with target cells via specific receptor-ligand interactions to mediate the transfer of proteins, bioactive lipids, mRNA, and miRNAs [21] to neighboring diseased or injured cells, thereby promoting the activation of regenerative programs [22, 23]. Previously, we demonstrated that the administration of kidney MSC-derived MPs (KMSC-MPs) repairs injured tissue and improves renal function via an mRNA-dependent mechanism in an experimental murine model of acute kidney injury. KMSC-MPs carried proangiogenic signals and ameliorated peritubular capillary rarefaction by inhibiting EMT in UUO kidneys, thus presenting an effective tool for stem cell-based therapy [24]. MPs have a superior safety profile to stem cell-based therapies as they have lower propensity to trigger immune responses and are unable to differentiate into tumor cells [22, 23]. In addition, MPs can be safely stored for extensive periods of time without losing their function [25]. However, our preliminary experiments using KMSC-derived MPs failed to demonstrate greater TGF-β-induced EMT inhibition in vitro or renal fibrosis improvements in UUO kidneys compared to direct rhEPO administration. Since MP characteristics depend on the cell of origin and the external stimuli or injury, recent studies have used preconditioning or gene modification to enhance the therapeutic efficacy of MSC-derived MPs [26]. In particular, MPs derived from hypoxia-preconditioned MSCs promoted angiogenesis more than those derived from MSCs cultured under normoxic conditions [27, 28], while gene overexpression enhanced the therapeutic efficacy of MSC-derived MPs in various disease models [29, 30].

Therefore, using MPs derived from EPO mRNA-overexpressing MSCs could be an appealing therapeutic strategy to effectively deliver EPO mRNA to target cells and promote tissue repair or regeneration. Here, we hypothesized that EPO mRNA-enriched MPs (hEPO-MPs) could be isolated from human EPO gene-transfected KMSCs (hEPO-KMSCs) and exert greater renoprotective effects than rhEPO. Consequently, we investigated whether hEPO-MP treatment could inhibit EMT in TGF-β1-treated Madin-Darby canine kidney (MDCK) cells in vitro and whether these inhibitory effects were mediated by changes in the Smad and p38 mitogen-activated protein kinase (MAPK) pathways. Moreover, we investigated whether hEPO-MPs had greater anti-fibrotic effects than MOCK-MPs in a murine model of UUO.

## Methods

### Human EPO mRNA-transfected KMSC generation and MP isolation

KMSCs isolated from an FVB male mouse kidney were cultured on gelatin-coated dishes in α-minimum essential medium (MEM) with 10% horse serum (Gem Biotech, Woodland, CA, USA) to 80% confluence, as previously described [31]. These KMSCs were transfected with the human EPO gene and used to generate MPs. Briefly, HEK293T cells (SeouLin Bioscience, Seongnam, South Korea), used as packaging cells, were transfected with a lentiviral expression vector containing *EPO*, *copGFP*, and packaging construct to generate lentiviral supernatants [32]. Meanwhile, empty control vectors were used to generate MOCK-KMSCs. After the KMSC cells had been transduced, hEPO-KMSCs were identified by cop green fluorescent protein (GFP) expression using fluorescence microscopy and EPO mRNA reverse transcription polymerase chain reaction (RT-PCR). The hEPO-KMSCs were then cultured on gelatin-coated dishes in MEM with 10% horse serum (Gem Biotech, Woodland, CA, USA), as described previously [31]. EPO secretion was measured in the supernatant of the hEPO-KMSCs by an enzyme-linked immunosorbent assay (ELISA) specific for human EPO (Quantikine, R&D Systems, Minneapolis, IL, USA).

To isolate hEPO-MPs, hEPO-KMSCs were cultured in serum-free α-MEM (Gibco, Carlsbad, CA, USA) and 1% O_2_ in a controlled atmosphere chamber using an O_2_ analyzer (Thermo Scientific, Marietta, OH, USA) for 24 h. Cell debris was removed by centrifugation at 2000×*g* for 10 min at room temperature and the cell-free supernatants were centrifuged at 50,000×*g* (Beckman Coulter Optima L-90 K Ultracentrifuge, CA, USA) for 2 h at 4 °C before being washed in phosphate-buffered saline (PBS) and centrifuged again under the same conditions. MOCK-MPs were isolated from the MOCK-KMSCs using the same protocol. hEPO-MP and MOCK-MP pellets were suspended in culture medium for the MDCK cell experiments and labeled with CellTracker™ (Invitrogen, Carlsbad, CA, USA) to allow them to be traced during in vivo and in vitro functional experiments.

### Characterization of isolated MPs

To analyze the characteristics of the isolated MPs, MP-containing pellets were resuspended in sterile 1× PBS or 1× Annexin V Binding Buffer (BD Biosciences, San Diego, CA, USA) consisting of 0.1 M HEPES/NaOH (pH 7.4), 1.4 M NaCl, and 25 mM CaCl2, and diluted 1:10 with distilled water for 1 h at 4 °C. Samples were kept on ice in the dark until flow cytometric analysis was performed using the following PE-conjugated antibodies: CD29, CD44, and CD73 (Biolegend, San Diego, CA, USA), as described previously with the BD FACS Canto II (BD Biosciences, San Jose, CA, USA). PE-conjugated isotype-matched antibodies were used as a negative control. Different sized beads (0.1, 0.2, 0.5, 1 μm, Invitrogen, Carlsbad, CA, USA) were used as size markers while a log scale was used to analyze forward (FSC) scatter and side (SSC) light scatter parameters. Spectral overlap compensation between fluorochrome channels was carried out for each experiment using single-color-stained MP populations. For each experimental sample, a corresponding isotype control or positive/negative MP populations were used to set gates. All data were analyzed using FlowJo software (TreeStar Inc., Ashland, OR, USA). Size distribution of the MPs was determined using nanoparticle tracking analysis (NanoSight NS300, Malvern Instruments, Malvern, UK).

### MDCK cell culture and treatment with rhEPO or MPs

To assess the effects of rhEPO and MPs in vitro, we used MDCK cells purchased from the Korean Cell Line Bank (Seoul, South Korea). The cells were seeded into six-well culture plates (5 × 10^4^ cells/well) with complete Dulbecco’s modified Eagle’s medium (DMEM) supplemented with 10% fetal bovine serum (FBS; Gibco, Carlsbad, CA, USA), penicillin G (100 U/mL), and streptomycin (100 U/mL) and cultured at 37 °C in 5% CO_2_ for 48 h. The cells were incubated in serum-free medium for 24 h and transferred into DMEM with 1% FBS (vehicle) or stimulated with recombinant human TGF-β1 (5 ng/mL, PeproTech, Rocky Hill, NJ, USA cat no. 100-21) for 48 h. The medium was changed and the cells were treated with TGF-β1 (5 ng/mL), MOCK-MPs (4 μg/mL), hEPO-MPs (4 μg/mL), or rhEPO (100 IU/mL) for a further 48 h. Incorporation of red fluorescent CellTracker™-labeled MPs into the cell cytoplasm was detected using a fluorescence microscope, and changes in MDCK cell morphology were assessed.

### Immunocytochemical staining of EMT markers

Treated MDCK cells were washed twice with PBS (pH 7.4), immediately fixed with 4% formaldehyde diluted in PBS for 15 min at room temperature, and permeabilized with 0.2% Triton X-100 for 10 min. The cells were then blocked with 1% bovine serum albumin for 30 min at room temperature, incubated overnight at 4 °C with the corresponding antibodies, and rhodamine-conjugated antibodies against rabbit IgG were used to detect the primary antibodies. Samples were stained with 4,6-diamidino-2-phenylindole (DAPI) and mounted using a Slow Fade Light Antifade kit with DAPI (Molecular Probes, Eugene, Oregon, USA). Immunostained cells were visualized using an LSM-780 META confocal microscope (Carl Zeiss, Oberkochen, Germany) at a wavelength of 405 nm for DAPI, 488 nm for GFP, and 647 nm for rhodamine. Fluorescence intensity was assessed by examining at least five fields per section under × 400 magnification by MetaMorph digital image analysis (BioVision, Exton, PA, USA).

### Immunoblotting of EMT markers and Smad/non-Smad proteins in MDCK cells

For western blotting, MDCK cells were lysed in 300 μL of cell lysis buffer containing 150 mM NaCl, 1% IGEPAL® CA-630, 0.5% sodium deoxycholate, 0.1% sodium dodecyl sulfate (SDS), 50 mM Tris (pH 8.0), and a protease inhibitor cocktail (Thermo Fisher Scientific, Waltham, MA, USA). Whole cell lysates were resolved by sodium dodecyl sulfate-polyacrylamide (SDS-PAGE) gel electrophoresis and transferred onto polyvinylidene fluoride membranes (Millipore, Billerica, MA, USA). After blocking with 5% skim milk, the membranes were incubated with primary antibodies against E-cadherin (1:500; BD Biosciences, Bedford, UK), α-SMA (1:10000; R&D, Minneapolis, IL, USA), fibronectin (1:1000; Santa Cruz, CA, USA), phospho-p38 (1:1000; Santa Cruz), p38 (1:1000; Abcam, Cambridge, UK), phospho-Smad2 (1:500; Millipore), Smad2 (1:1000; Santa Cruz), phospho-Smad3 (1:500; Abcam), Smad3 (1:1000; Cell Signaling Technology, Danvers, MA, USA), and GAPDH (1:1000; Cell Signaling Technology, Danvers, MA, USA). The membranes were then washed three times in 1× PBS with Tween-20 (VWR Amresco, Solon, Ohio, USA) for 5 min and incubated with horseradish peroxidase-conjugated secondary antibodies. Target proteins were visualized using Amersham ECL Western Blotting Detection Reagent (GE Healthcare Life Sciences). Band densities were measured using NIH Image J software.

### Quantitative real-time polymerase chain reaction (qRT-PCR) of MDCK cells

Total RNA was isolated from MDCK cells using an RNeasy Mini Kit (QIAGEN, Germantown, MD, USA) and cDNA was synthesized using a DNA Synthesis Kit (QIAGEN). qRT-PCR was performed using an ABI StepOnePlus real-time PCR system (Applied Biosystems, Beverly, MA, USA) with double-stranded DNA synthesis monitored using SYBR Green (Applied Biosystems). Triplicate test reactions were carried out for each sample to analyze gene expression, which was normalized to β-actin mRNA expression. Negative cDNA controls were cycled in parallel with each run. The primer sequences are listed in Table 1.

**Table 1:** qRT-PCR primers

Gene	Forward primers (5′ to 3′)	Reverse primers (5′ to 3′)
Canine E-cadherin	CTGTCACTGTGGACGTGGAA	CTTGCGCCGTGTGTTAGTTCC
Canine vimentin	AACCGGAACAATGATGCCCT	CATTTCACGCATCTGGCGTT
Canine α-SMA	ATGCAGAAGGAGATCACCGC	CACAGAGCAAGGAAGCGTCT
β-actin	CGCAATGAAGTGGAGTCTGA	ATAGCAGCAGACAGAGGCAAC

### Animal experiments

Adult FVB/N mice (five to seven weeks old) were purchased from Koatech (Gyeonggi-Do, Korea). The animals were maintained under temperature-controlled conditions with a 12-h light/dark cycle and were provided with water and food ad libitum. UUO was performed using an established protocol [24]. Briefly, FVB mice were anesthetized with isoflurane plus oxygen and placed on a heating pad (Jeung Do Bio & Plant Co, Seoul, Korea) to maintain their temperature at 37 °C. The left ureter was exposed using a flank incision and ligated with 3–0 silk at two points just below the lower pole of the left kidney, and then, the peritoneal membrane and skin were sutured. Sham animals underwent the same procedure, except that the ureter was not ligated. Following surgery, the mice were randomly divided into four groups and administered the vehicle (150 μL saline) only, MOCK-MPs (80 μg/150 μL), or hEPO-MPs (80 μg/150 μL) via the tail vein (*n* = 5 per group). In the rhEPO-treated group, rhEPO (1000 U/kg) was administered intraperitoneally every other day. Our previous study showed that 7 days of UUO could induce significant TIF in FVB/N mice [24]. Therefore, all mice were sacrificed 7 days after UUO surgery, and the unilaterally obstructed kidney was harvested for tissue collection. Half of the kidney tissue was fixed in 4% paraformaldehyde (PFA) for subsequent histology and immunofluorescence, while the remaining half was flash frozen for protein and mRNA isolation.

### Immunohistochemical and immunofluorescence analysis

To analyze kidney fibrosis, kidney sections were stained with Sirius red, Masson’s trichrome, and anti-α-SMA (Sigma) and examined by light microscopy. Positive Sirius red, Masson’s trichrome, and anti-α-SMA (Sigma) staining areas were evaluated relative to the unit area and expressed as a percentage per unit area using MetaMorph microscopy image analysis software (Molecular Devices, Sunnyvale, CA, USA). Macrophage immunohistochemistry was performed using anti-F4/80 (Abcam) antibodies and F4/80-positive cells quantified as the number of cells per high power field [24]. Microscopy assessment was carried out in a blinded manner, with 20 randomly selected fields from each slide section examined at × 400 magnification. To trace MPs in vivo, freshly isolated MPs were labeled with red fluorescent CellTracker™ and detected within the peritubular interstitium of kidney sections by immunofluorescence microscopy.

### Immunoblotting of renal fibrosis markers

For western blotting, mouse kidney tissues were lysed in 300 μL of cell lysis buffer containing 150 mM NaCl, 1% IGEPAL® CA-630, 0.5% sodium deoxycholate, 0.1% sodium dodecyl sulfate (SDS), 50 mM Tris (pH 8.0), and a protease inhibitor cocktail (Thermo Fisher Scientific). Kidney tissue lysates were resolved by sodium dodecyl sulfate-polyacrylamide (SDS-PAGE) gel electrophoresis and transferred onto polyvinylidene fluoride membranes (Millipore), blocked with 5% skim milk, and incubated with primary antibodies against E-cadherin (1:1000; Santa Cruz), collagen I (1:500; Santa Cruz), α-SMA (1:10000; R&D), fibronectin (1:1000; Santa Cruz), TGF-β1 (1:1000; Santa Cruz), and GAPDH (1:1000; Cell Signaling Technology). The membranes were then washed three times with 1× PBS with Tween-20 (AMRESCO®) for 5 min, incubated with horseradish peroxidase-conjugated secondary antibodies, and washed again using the same procedure. Target proteins were visualized using Amersham ECL Western Blotting Detection Reagent (GE Healthcare Life Sciences), and band density was measured using NIH Image J software.

### Statistical analysis

Data were expressed as the mean ± standard deviation and were statistically analyzed using SPSS 18.0 software (SPSS Inc., Chicago, IL, USA). Between-group comparisons were made by one-way analysis of variance (ANOVA) followed by the Student-Newman-Keuls test. Multiple tests were applied only when a significant difference was determined by ANOVA. *P* values of < 0.05 were considered statistically significant. All experiments were repeated at least three times with similar results.

## Results

### hEPO-KMSC generation and hEPO-MP characterization

**Fig. 1:**
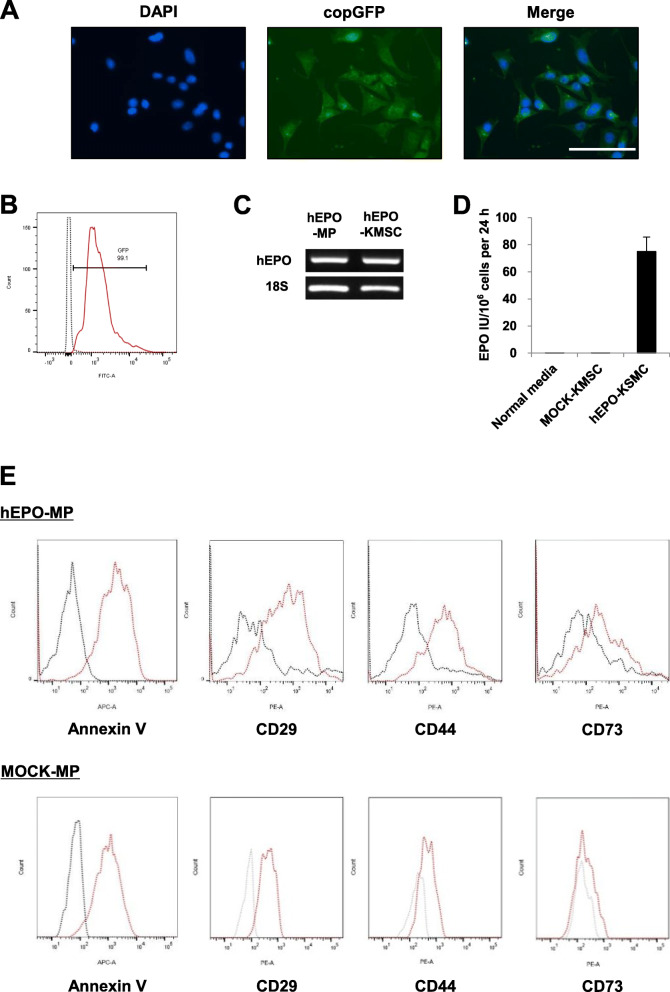
hEPO-KMSC generation and hEPO-KMSC and hEPO-MP characterization. **a** Fluorescence microscopy revealed that hEPO-KMSCs expressed copGFP and **b** flow cytometry demonstrated efficient transfection. **c** RT-PCR detection of hEPO mRNA in hEPO-KMSCs and hEPO-MPs. **d** hEPO secretion by hEPO-KMSCs measured by ELISA on unconditioned culture media or 24 h conditioned media from MOCK- or hEPO-KMSCs. **e** Flow cytometry analysis of cell surface marker (CD29, CD44, CD73) and Annexin V expression on hEPO-MPs (upper panel) and MOCK-MPs (lower panel). Red lines indicate CD29, CD44, CD73, and Annexin V expression. Black lines indicate isotypic controls. Data are representative of three independent experiments. Magnification, × 400. Scale bar, 100 μm (white)

Cultured hEPO-KMSCs were identified following lentiviral transduction by copGFP expression using fluorescence microscopy (Fig. 1a), with flow cytometric analysis revealing a transfection efficiency of > 99% based on the percentage of copGFP-expressing cells (Fig. 1b). In addition, hEPO mRNA expression was confirmed by RT-PCR with cDNA isolated from hEPO-KMSCs and hEPO-MPs (Fig. 1c, right and left panels, respectively). In vitro EPO secretion was measured in 24 h conditioned supernatant, revealing that MOCK-KMSCs did not secrete detectable levels of EPO, whereas hEPO-KMSCs produced ~ 75 IU of EPO per 1 × 10^6^ cells (Fig. 1d). On average, the MPs were 225.5 nm in size, while both hEPO-MPs and MOCK-MPs were positive for Annexin V (> 90%) and expressed CD29, CD44, and CD73, similarly to their cells of origin (Fig. 1e and Supplementary Fig. 1).

### hEPO-MP treatment suppressed TGF-β1-induced EMT in MDCK cells

**Fig. 2:**
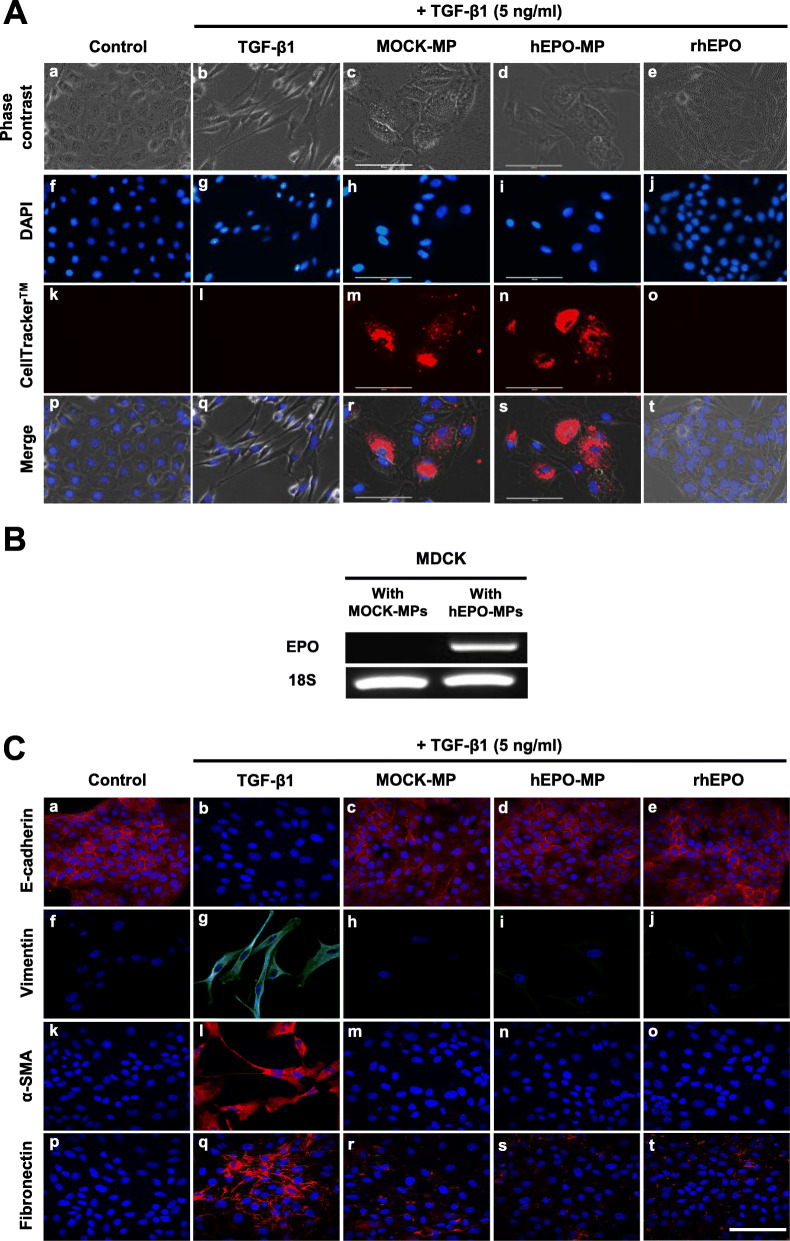
MPs and rhEPO attenuate TGF-β1-induced EMT in MDCK cells. **A** Morphological changes in TGF-β1-treated MDCK cells observed by phase contrast microscopy. Compared to control MDCK cells (a), TGF-β1 treatment changed MDCK cell morphology from cuboidal to an elongated spindle-like shape (b). These morphological changes were reversed by co-treatment with MOCK-MPs (c), hEPO-MPs (d), and rhEPO (e). Fluorescence microscopy of red fluorescent CellTracker™-labeled MP incorporation into MDCK cells (m, n, r, s). Blue staining represents nuclear counterstaining with DAPI (f–j, p–t). **B** RT-PCR of hEPO mRNA expression in MDCK cells co-treated with hEPO-MPs, demonstrating that MPs mediated the horizontal transfer of hEPO mRNA into target MDCK cells. **C** Immunofluorescence microscopy of E-cadherin, vimentin, α-SMA, and fibronectin in MDCK cells after co-treatment with TGF-β1 and MPs or rhEPO, indicating reduced E-cadherin immunostaining intensity in response to TGF-β1-treatment (b) and its reversal by co-treatment with MOCK-MPs (c), hEPO-MPs (d), and rhEPO (e). TGF-β1 treatment increased vimentin (g), α-SMA (l), and fibronectin (q) immunostaining intensity which was reversed by co-treatment with MOCK-MPs (h, m, r), hEPO-MPs (i, n, s), and rhEPO (j, o, t). Magnification, × 400. Scale bars, 100 μm (white)

To evaluate whether hEPO-MP treatment could affect TGF-β1-induced EMT, we examined the effect of hEPO-MPs on the expression of EMT markers in MDCK cells. MDCK cells were cultured with 5 ng/mL of TGF-β1 for 48 h to induce EMT, as confirmed by a change in the morphology of TGF-β1-treated cells from cuboidal to an elongated spindle shape (Fig. 2A a, b, p, q). Following treatment with CellTracker™-labeled hEPO-MPs, their incorporation into the cytoplasm of MDCK cells was observed (Fig. 2A n, s) alongside the direct transfer of EPO mRNA into target MDCK cells, as demonstrated by RT-PCR (Fig. 2B). Moreover, co-treatment of TGF-β1-treated MDCK cells with MOCK-MPs, hEPO-MPs, or rhEPO reversed TGF-β1-induced morphological changes (Fig. 2A c–e).

**Fig. 3:**
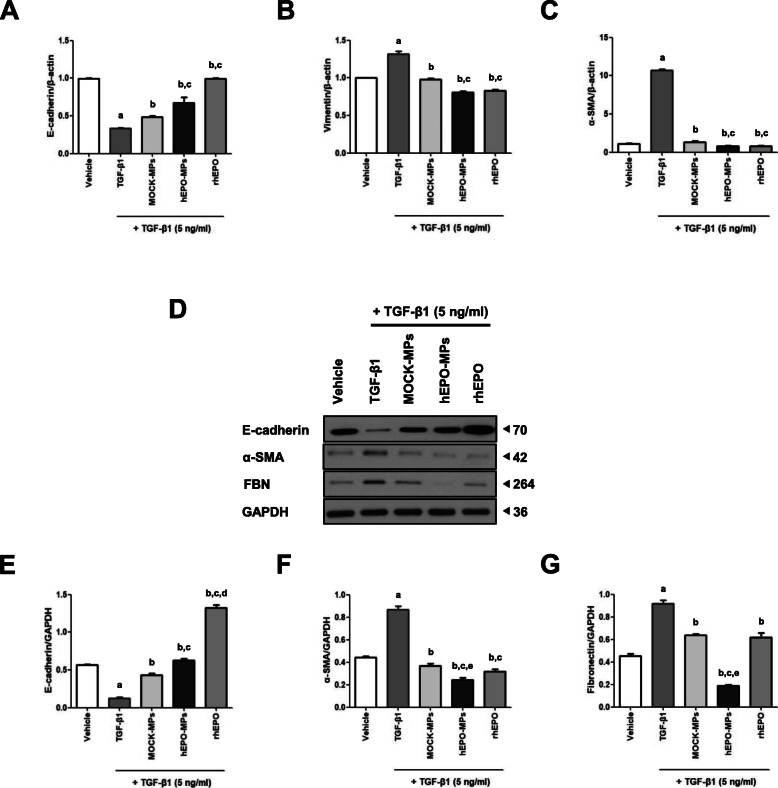
Effect of MPs or rhEPO on EMT markers in MDCK cells. **A**–**C** qRT-PCR of E-cadherin, vimentin, and α-SMA mRNA in MDCK cells co-treated with TGF-β1 and MPs or rhEPO. Compared to vehicle-treated MDCK cells, TGF-β1 treatment significantly decreased E-cadherin and increased vimentin and α-SMA mRNA expression. MOCK-MPs, hEPO-MPs, and rhEPO reversed these changes in E-cadherin, vimentin, and α-SMA mRNA expression. **D** Western blotting for E-cadherin, α-SMA, and fibronectin protein levels in MDCK cells co-treated with TGF-β1 and MPs or rhEPO. Compared to vehicle-treated MDCK cells, TGF-β1 significantly decreased E-cadherin expression (**E**) and increased α-SMA and fibronectin expression (**F**, **G**). Co-treatment with MOCK-MPs, hEPO-MPs, and rhEPO significantly ameliorated these changes induced by TGF-β1. Western blotting data were normalized to GAPDH. Molecular weights are shown in kDa. Data are presented as mean ± SD; *n* = 4~5 for each experimental group. ^a^*P* < 0.05 vs. vehicle, ^b^*P* < 0.05 vs. TGF-β1, ^c^*P* < 0.05 vs. TGF-β1+MOCK-MPs, ^d^*P* < 0.05 vs. TGF-β1+hEPO-MPs, ^e^*P* < 0.05 vs. TGF-β1+rhEPO

Changes in E-cadherin and vimentin expression are the most important features of EMT. In this study, we examined the expression of EMT-related factors E-cadherin, fibronectin, vimentin, and α-SMA accompanying changes in MDCK cell morphology, using immunofluorescence staining (Fig. 2C). E-cadherin expression was significantly lower in TGF-β1-treated MDCK cells than in the control MDCK cells, whereas vimentin, α-SMA, and fibronectin expression levels were higher. Treatment with MOCK-MPs, hEPO-MPs, or rhEPO increased E-cadherin expression on the cell membrane and decreased vimentin, α-SMA, and fibronectin expression in the cytoplasm. Consistent with the observed changes in cell morphology, TGF-β1 treatment significantly downregulated E-cadherin and upregulated α-SMA and vimentin mRNA expression (Fig. 3A–C), while co-treatment with MOCK-MPs, hEPO-MPs, or rhEPO ameliorated these changes. Notably, E-cadherin expression was significantly higher in the hEPO-MP or rhEPO-treated groups than in those treated with MOCK-MPs; however, MOCK-MPs, hEPO-MPs, and rhEPO all significantly and comparably reduced α-SMA and vimentin mRNA expression.

Immunoblot analysis also demonstrated that TGF-β1 significantly decreased E-cadherin and increased α-SMA and fibronectin protein levels in MDCK cells. Moreover, these changes were effectively reversed by treatment with MOCK-MPs, hEPO-MPs, or rhEPO (Fig. 3D–G). In particular, hEPO-MPs decreased α-SMA and fibronectin expression more effectively than MOCK-MPs or rhEPO, whereas hEPO-MPs and rhEPO significantly increased E-cadherin expression compared to MOCK-MPs.

### hEPO-MPs attenuate TGF-β1-induced upregulation of Smad2, Smad3, and p38 MAPK phosphorylation in MDCK cells

**Fig. 4:**
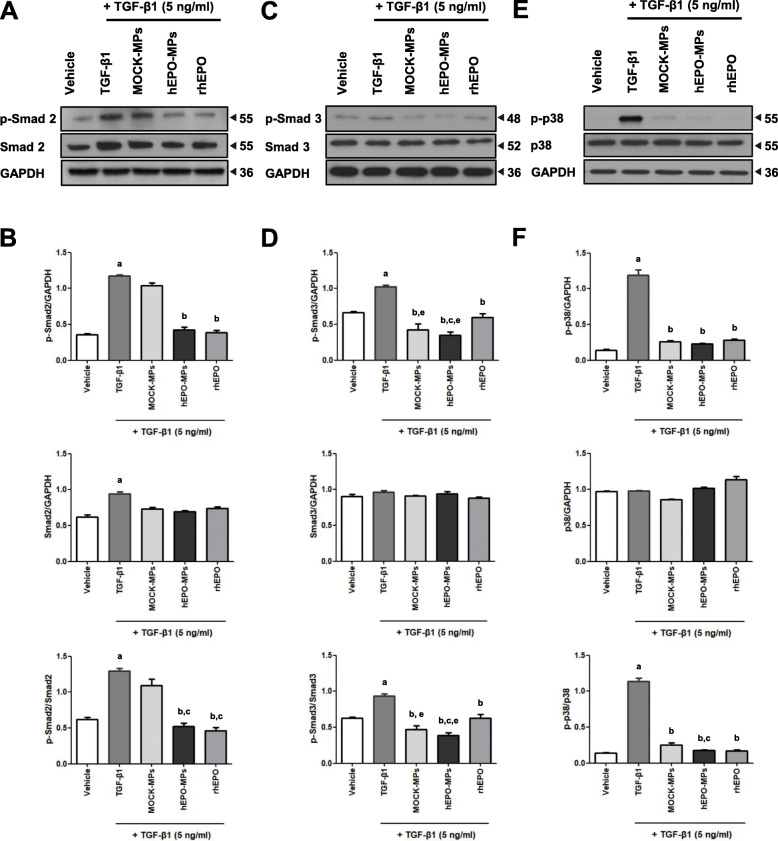
Effect of MPs and rhEPO on Smad2, Smad3, and p38 MAPK in MDCK cells. TGF-β1 significantly increased phosphorylated Smad2/Smad2 and phosphorylated Smad3/Smad3 levels (**A**–**D**) as well as phosphorylated p38/p38 (**E**, **F**) levels. hEPO-MPs and rhEPO significantly attenuated TGF-β1-induced phosphorylated Smad2/Smad2 upregulation, whereas MOCK-MPs did not (**A**, **B**). MOCK-MPs, hEPO-MPs, and rhEPO significantly inhibited TGF-β1-induced phosphorylated Smad3/Smad3 and phosphorylated p38/p38 upregulation with hEPO-MPs showing greatest effect (**C**–**F**). Protein expression was calculated using NIH Image J software. Western blot data were normalized to GAPDH. Molecular weights are shown in kDa. Data are presented as the mean ± SD; *n* = 4~5/for each experimental group. ^a^*P* < 0.05 vs. vehicle, ^b^*P* < 0.05 vs. TGF-β1, ^c^*P* < 0.05 vs. TGF-β1+MOCK-MPs, ^e^*P* < 0.05 vs. TGF-β1+rhEPO

Previous studies have demonstrated that rhEPO attenuates the upregulation of Smad2 phosphorylation and inhibits EMT in TGF-β1-treated MDCK cells. Here, we investigated whether hEPO-MP treatment also modulates TGF-β1-induced EMT by inhibiting the Smad signaling pathway. Phosphorylated Smad2 and Smad3 (p-Smad2 and p-Smad3, respectively) protein levels were analyzed in MDCK cells by immunoblotting (Fig. 4A–D), revealing that both p-Smad2 and p-Smad3 expression, as well as the p-Smad2 to Smad2 and p-Smad3 to Smad3 ratios were significantly higher in TGF-β1-treated MDCK cells than in the control MDCK cells. Consistent with our previous findings, a single dose of hEPO-MPs significantly reduced p-Smad2 expression and the p-Smad2 toSmad2 ratio in a manner comparable to rhEPO. Conversely, MOCK-MPs failed to significantly decrease p-Smad2 expression or the p-Smad2 toSmad2 ratio but reduced p-Smad3 expression and the p-Smad3 to Smad3 ratio. Of note, MOCK-MPs reversed TGF-β1-mediated changes in EMT markers in MDCK cells. TGF-β1-mediated EMT is known to be regulated by pathways other than Smad signaling, including the p38 MAPK signaling pathway, which is induced by TGF-β1 [33]. Therefore, we investigated whether rhEPO or MP treatment affected p38 MAPK signaling in TGF-β1-induced EMT by measuring phosphorylated p38 (p-p38) MAPK and p38 MAPK expression in TGF-β1-treated MDCK cells (Fig. 4E, F). Stimulation with TGF-β1 markedly increased p-p38 MAPK expression compared to the control, while MOCK-MP, rhEPO-MP, and rhEPO treatment significantly suppressed p-p38 MAPK expression in TGF-β1-mediated EMT.

### hEPO-MP treatment ameliorated renal fibrosis in UUO kidneys

**Fig. 5:**
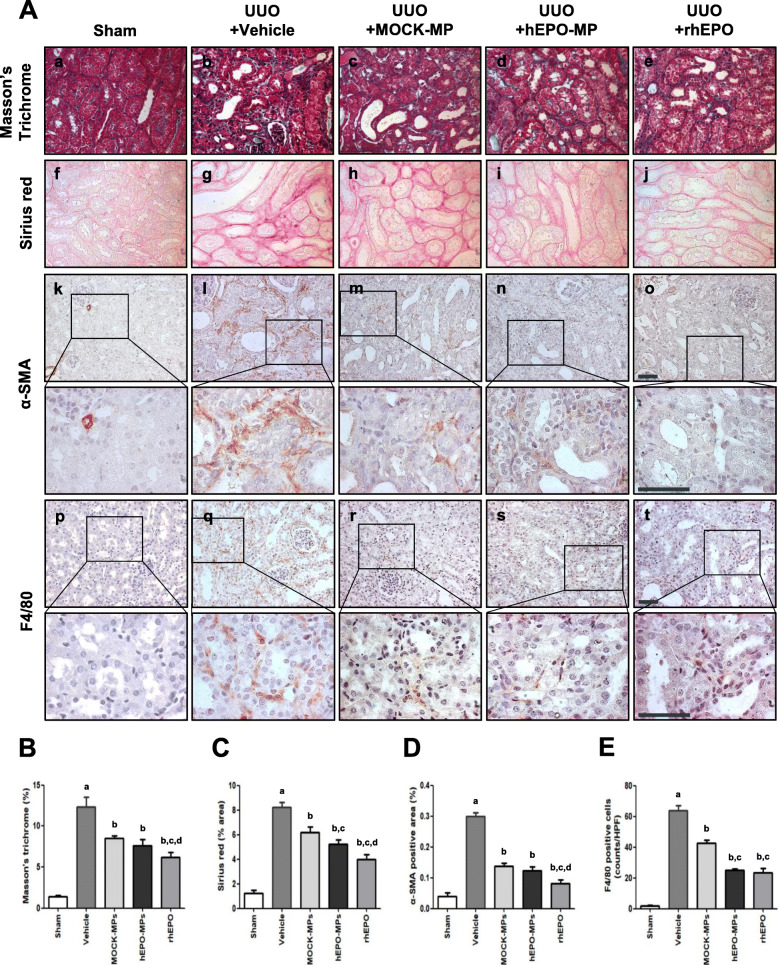
Anti-fibrotic effects of MPs or rhEPO in UUO kidneys. **A** Microscopic analysis of Masson’s trichrome and Sirius red-stained UUO kidney sections. Sham-operated control (a, f), obstructed kidney treated with vehicle control (b, g), and UUO kidneys treated with MOCK-MPs (c, h), hEPO-MPs (d, i), and rhEPO (e, j). MPs and rhEPO significantly attenuated renal interstitial fibrosis as quantified using Image J and MetaMorph software (**B**, **C**). Immunohistochemical analysis of α-SMA expression in sham-operated control (k) and UUO kidneys treated with the vehicle control (l), MOCK-MPs (m), hEPO-MPs (n), and rhEPO (o). MPs and rhEPO significantly attenuated α-SMA expression in obstructed kidneys (**D**). Immunohistochemical analysis of F4/80-positive cells in sham-operated control (p) and UUO kidneys treated with vehicle control (q), MOCK-MPs (r), hEPO-MPs (s), and rhEPO (t). MPs and rhEPO significantly attenuated the infiltration of F4/80-positive macrophage/inflammatory cells in obstructed kidneys. Quantitative assessment of renal F4/80-positive macrophage/inflammatory cells (**E**). Magnification, × 400 and × 1000 (for inserts). Scale bars, 50 μm (black). Data are presented as the mean ± SD; *n* = 5 for each experimental group. ^a^*P* < 0.05 vs. sham control, ^b^*P* < 0.05 vs. UUO+vehicle, ^c^*P* < 0.05 vs. UUO+MOCK-MPs, ^d^*P* < 0.05 vs. UUO+hEPO-MPs

To assess the beneficial effects of hEPO-MPs on renal fibrosis, we used an UUO mouse model. First, we examined whether CellTracker™-labeled MPs were delivered into the kidneys after their injection via the tail vein, and found labeled MPs selectively in the interstitial areas of the UUO kidneys in vivo (Supplement Fig. 2). Next, we investigated the degree of renal TIF in UUO kidneys with Sirius red and Masson’s trichrome staining; vehicle-treated UUO kidneys showed significantly higher collagen expression than the sham-operated kidneys. Although both MPs and rhEPO significantly reduced collagen deposition in the UUO kidneys, the kidneys treated with hEPO-MP and rhEPO showed a greater decrease in TIF than the MOCK-MP-treated kidneys (Fig. 5A a–j, B, C).

### hEPO-MP treatment decreased UUO-induced renal myofibroblast accumulation in renal tissue

Myofibroblasts are an activated fibroblast phenotype that are responsible for extracellular matrix deposition during TIF and are recognized as an important factor in disease progression. Immunohistochemical staining for the myofibroblast marker α-SMA and quantitative analysis of α-SMA-positive areas revealed that the α-SMA-positive area was almost 6-fold higher in the UUO experimental group than in the sham-operated control group. However, treatment with rhEPO or both MPs significantly decreased the α-SMA-positive area in UUO kidneys, particularly those treated with rhEPO (Fig. 5A k–o, D), while hEPO-MPs and MOCK-MPs yielded comparable reductions.

### hEPO-MP treatment reversed EMT markers and renal fibrosis in UUO kidneys

**Fig. 6:**
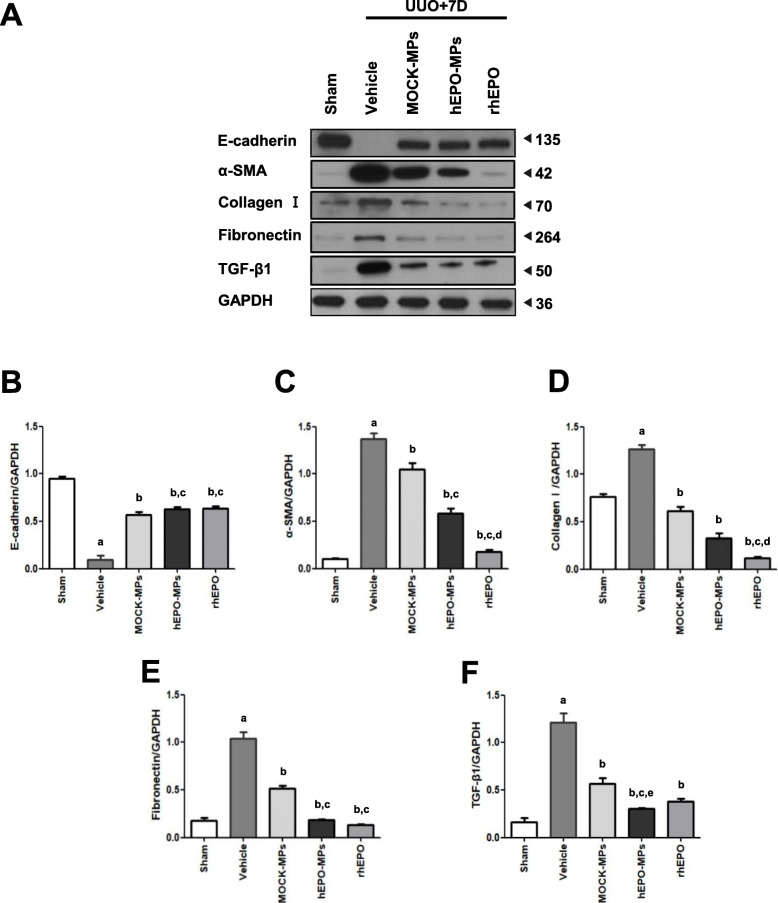
Western blotting of α-SMA, collagen I, fibronectin, and TGF-β1 in UUO kidneys. Compared to the sham-operated control, the obstructed kidneys of vehicle-treated mice showed significantly lower E-cadherin (**B**) and higher α-SMA (**C**), collagen I (**D**), fibronectin (**E**), and TGF-β1 (**F**) expression. Treatment with MOCK-MPs, hEPO-MPs, and rhEPO significantly reversed these changes. Protein levels were calculated using NIH Image J software. Western blot data were normalized to GAPDH. Molecular weights are shown in kDa. Data are presented as the mean ± SD; *n* = 5 for each experimental /group. ^a^*P* < 0.05 vs. sham control, ^b^*P* < 0.05 vs. UUO+vehicle, ^c^*P* < 0.05 vs. UUO+MOCK-MPs, ^d^*P* < 0.05 vs. UUO+hEPO-MPs, ^e^*P* < 0.05 vs. UUO+rhEPO

Next, we investigated changes in EMT (α-SMA and E-cadherin) and renal fibrosis (collagen I and TGF-β1) markers in UUO kidneys. Western blot analysis of vehicle-treated UUO kidneys revealed significant increases in α-SMA, collagen I, and TGF-β1 expression compared to that in sham-operated control kidneys (Fig. 6). MOCK-MPs, hEPO-MPs, and rhEPO reversed these changes, consistent with the results of the in vitro experiments using MDCK cells. In particular, rhEPO treatment most effectively reduced α-SMA and collagen I levels in UUO kidneys. Conversely, E-cadherin expression was significantly decreased in UUO kidneys but increased with rhEPO, MOCK-MP, and hEPO-MP treatment. Taken together, these findings indicate the renoprotective effects of MPs in vivo.

### hEPO-MP treatment ameliorates UUO-induced renal inflammatory cell infiltration

To further investigate the anti-fibrotic effects of hEPO-MPs, we assessed F4/80-positive macrophage infiltration into the tubulointerstitial area of UUO kidneys. Semi-quantitative MetaMorph analysis revealed that infiltration of F4/80-positive macrophages increased significantly in vehicle-treated UUO kidneys but decreased significantly when the kidneys were treated with hEPO-MPs, MOCK-MPs, and rhEPO (71.5 ± 0.08%, 44.8 ± 0.03%, and 36.5 ± 0.05% of vehicle control; Fig. 5A p–t, E). In particular, hEPO-MPs and rhEPO reduced F4/80-positive cell infiltration by 2-fold more than MOCK-MPs, suggesting that hEPO-MPs have a significantly greater capacity to inhibit F4/80-positive macrophage infiltration than rhEPO.

## Discussion

Recent studies have suggested that EMT of renal tubular epithelial cells is a major contributor to myofibroblasts and fibroblasts during renal fibrogenesis [34, 35]; thus, inhibiting or improving EMT could be a valuable therapeutic strategy for inhibiting renal fibrosis. Moreover, studies have demonstrated that EPO has cytoprotective effects, while rhEPO treatment inhibits EMT in renal TECs. In this study, we investigated whether hEPO-MPs could improve MSC-based renal fibrosis treatment. Our findings revealed that hEPO-MPs were associated with the direct transfer of EPO mRNA to target cells and inhibited TGF-β1-induced EMT in MDCK cells by regulating p-Smad and p-p38 MAPK signaling. Moreover, hEPO-MPs reduced TGF-β1 and EMT marker expression and inhibited inflammatory macrophage infiltration in UUO kidneys, suggesting that hEPO-MP treatment may inhibit renal fibrosis.

Preclinical studies have shown that stem cell-based therapies could be a promising novel therapeutic strategy to slow the progression of renal diseases; in particular, MSC-derived MPs have shown beneficial effects in models of acute kidney injury or CKD, as indicated by improved histological outcomes and functional parameters [36]. MPs released by MSCs can induce the same paracrine effects as MSC therapy to regulate growth factors or cytokines and ameliorate renal inflammation and fibrosis [37, 38]. Previously, we demonstrated that KMSC-MPs deliver proangiogenic genes to target cells to promote robust improvement in renal injury [39]. These beneficial paracrine effects also involve factors related to EMT, which may explain why MOCK-MPs also significantly improved EMT and renal fibrosis. MSC-derived MPs are also more clinically useful than MSCs as they circumvent concerns regarding extensive MSC expansion or maldifferentiation in target tissues.

The therapeutic potential of MSC-derived MPs largely depends on the nature of the original MSCs since MPs isolated from different cells have heterogeneous mRNA contents [40] and thus exert different therapeutic effects under different disease conditions. It would therefore be appealing to combine specific growth factors with regenerative effects against renal TECs or endothelial cells with an MSC therapeutic platform. In this study, we compared the anti-fibrotic and inhibitory effects of MOCK-MPs and rhEPO in TGF-β-induced EMT. Previous studies have successfully used whole MOCK-MPs, which contain all factors derived from MSCs, as a novel therapeutic platform for acute kidney injury [22, 39]. In this study, MOCK-MPs reduced TGF-β-induced EMT and improved renal fibrosis in UUO kidneys; however, the beneficial effects were significantly lesser than those of rhEPO treatment.

EPO is the major growth factor involved in erythropoiesis and has been used to treat renal anemia in patients with advanced CKD in clinical practice [41]. Studies have demonstrated that EPO has cytoprotective effects in many non-erythroid cells, including renal TECs, which are largely attributed to anti-apoptosis, anti-oxidant, and anti-inflammatory effects [42]. Moreover, EPO has been shown to upregulate miR-200 expression and attenuate hypoxia-induced EMT in HK-2 cells [43] as well as counteract TGF-β1-induced EMT by regulating TGF-β/Smad-2 signaling and inhibit renal fibrosis in UUO kidneys [15]. Unfortunately, repetitive high-dose rhEPO treatments were required to achieve these benefits, which would translate to ~ 210,000 U in a 70-kg patient, approximately 10 times the dose normally administered to patients with severe renal anemia. Such high doses have been proven clinically unsafe and may increase cardiovascular complications [44], thus raising concerns regarding the clinical implementation of high-dose EPO to treat renal fibrosis.

Generation of EPO-producing MSCs by genetic engineering has been reported to enhance the benefits of MSC-based treatments in cardiovascular disease by improving their promotion of the angiogenic response and survival potential via endocrine and autocrine mechanisms, respectively [45]. In this study, we genetically engineered KMSCs to overexpress EPO mRNA and secrete hEPO in culture. Upon hypoxic stimulation, hEPO-KMSCs readily secreted MPs that were enriched with hEPO mRNA. As expected, co-culturing MDCK cells with these isolated hEPO-MPs resulted in the horizontal transfer of hEPO mRNA into recipient MDCK cells and inhibited TGF-β-induced EMT. Previous studies have demonstrated that rhEPO treatment has potential effects on Smad signaling pathways in EMT as well as renal fibrosis [15]. We further investigated the effects of hEPO-MPs on the TGF-β/non-Smad pathways during TGF-β1-induced EMT in MDCK cells. Compared to rhEPO, hEPO-MPs treatment displayed comparable inhibition of Smad2 phosphorylation and greater inhibition of Smad3 phosphorylation in TGF-β1-induced EMT in vitro. In contrast, MOCK-MPs failed to significantly decrease Smad2 phosphorylation. Moreover, significant p-p38 MAPK overexpression was noted in TGF-β-induced EMT, consistent with the known role of the p38 MAPK pathway in renal fibrosis, acting downstream of the TGF-β1 pathway. Unlike Smad2, both MPs effectively inhibited p-p38 MAPK overexpression, with hEPO-MPs again showed a stronger effect than MOCK-MPs, supporting the beneficial effects of hEPO-MP administration. Although the exact significance of this finding requires further investigation, it is evident that hEPO-MPs strongly suppress the pSmad2, pSmad3, and p38 MAPK signaling pathways in a manner comparable to rhEPO. Both hEPO-MPs and MOCK-MPs inhibited renal fibrosis in UUO kidneys, but hEPO-MPs showed greater anti-fibrotic effect than the MOCK-MPs. Noteworthily, in this study, repeated administration of high rhEPO doses was needed to ameliorate renal fibrosis, rather than a single infusion of hEPO-MPs. Previous MSC therapies have reported the selective homing of administered MSCs to the site of injury [22, 39], consistent with our results showing the preferential engraftment of hEPO-MPs and their EPO mRNA contents to the inflammatory site of kidney injury, making this delivery platform highly attractive. Gene overexpression has been shown to enhance the therapeutic efficacy of MSC-derived MPs in various disease models [29, 30]; therefore, using hEPO-MPs as a therapeutic strategy may be more convenient as well as physiologically relevant than systemic rhEPO administration. To our knowledge, our findings are the first to demonstrate that genetically modified hEPO-MPs could be used as a novel therapeutic agent to inhibit renal fibrosis.

Macrophages are highly involved in the process of renal injury and contribute toward the development of renal fibrosis. In this study, macrophage depletion markedly reduced myofibroblast formation and TIF in UUO kidneys, while interstitial myofibroblast proliferation has been shown to lead to excessive extracellular matrix deposition and increased renal fibrosis [46]. Moreover, EPO administration in an animal model of CKD was found to suppress EMT and inflammatory cell infiltration, thus reducing renal fibrosis [15]. These beneficial effects may be partly explained by the anti-inflammatory effects of EPO observed in various ischemia-reperfusion injury models [47]. Consistent with previous studies, we showed that MPs can inhibit myofibroblast activation and reduce F4/80-positive cell infiltration in obstructed kidneys. In fact, hEPO-MPs were twice as efficient at blocking the recruitment of F4/80 inflammatory cells in UUO kidneys, which may contribute toward improved renal fibrosis.

The modified EPO gene-transfected MSC-based therapy described herein is a promising therapeutic strategy for treating CKD; however, there are several issues that need to be resolved before it can be implemented widely. Firstly, MPs derived from gene-modified MSCs may have the potential to induce target cells to undergo malignant transformation, raising biosafety concerns [45], even though MPs are acellular, lack proliferative capacity, and have lower tumorigenic potential than MSCs. No studies have yet examined the long-term effects of genetically modified MSC-derived MPs, and further studies are required to address this important issue. Secondly, there is uncertainty regarding the optimal therapeutic dose regimen or route of administration for MSC-derived MPs. In this study, we investigated the benefits of hEPO-MPs using a fixed dose regimen described in our previous studies [24, 39]; however, the in vivo efficacy of increasing MP doses or a threshold dose in experimental renal disease have not yet been reported. Dose and injection intervals would influence the ability of MPs to home and engraft damaged target cells, thereby increasing their therapeutic efficacy. Future studies should therefore determine the adequate number of MP injections and optimal intervals between therapies. Finally, there are issues regarding the isolation and quantification of MPs by different investigators. Although recent methodological guidelines have been published to guide the proper isolation, handling, and storage of MPs [48], further studies are required to determine whether renal outcomes vary according to protocols.

## Conclusions

Our study found that hEPO-MPs contribute toward the inhibition of TGF-β1-induced EMT in MDCK cells in vitro and improve renal fibrosis in obstructed kidneys in vivo in a similar manner to that of rhEPO; thus, they may be used as a novel therapeutic agent.

## Supplementary information

**Additional file 1: Fig. S1**. MOCK and hEPO-KMSC characterization. Flow cytometry analysis of cell surface marker expression on cells. Red lines indicate CD29, CD44, CD45 and CD73 expression. Black lines indicate isotypic controls. **Fig. S2.** Fluorescence microscopy of frozen obstructed kidney tissue sections showing red CellTracker™-labeled MPs colocalized within the interstitium. White arrows indicate red CellTracker™-labeled MPs. **Fig. S3.** RT-PCR analysis of hEPO mRNA expression. Compared to hEPO-MPs, MOCK-MPs failed to induce hEPO mRNA expression. (PPTX 1940 kb)

## Data Availability

The datasets used and/or analyzed during the current study are available.

## Abbreviations

α-SMA: Alpha-smooth muscle actin
CKD: Chronic kidney disease
EPO: Erythropoietin
ECM: Extracellular matrix
EMT: Epithelial-to-mesenchymal transition
KMSC: Kidney-derived MSC
MDCK: Madin-Darby canine kidney cells
MSC: Mesenchymal stem cell
MP: Microparticle
TEC: Tubule epithelial cell
TGF-β1: Transforming growth factor-beta1
TIF: Tubulointerstitial fibrosis
UUO: Unilateral ureteral obstruction
