# Supplementary material for: Microparticles derived from human erythropoietin mRNA-transfected mesenchymal stem cells inhibit epithelial-to-mesenchymal transition and ameliorate renal interstitial fibrosis
Source: Stem Cell Res Ther. 2020 Sep 29;11:422. doi: 10.1186/s13287-020-01932-z (PMC7523343; doi:10.1186/s13287-020-01932-z)
Supplement: Supplementary file 1 — Additional file 1: Fig. S1. MOCK and hEPO-KMSC characterization. Flow cytometry analysis of cell surface marker expression on cells. Red lines indicate CD29, CD44, CD45 and CD73 expression. Black lines indicate isotypic controls. Fig. S2. Fluorescence microscopy of frozen obstructed kidney tissue sections showing red CellTracker™-labeled MPs colocalized within the interstitium. White arrows indicate red CellTracker™-labeled MPs. Fig. S3. RT-PCR analysis of hEPO mRNA expression. Compared to hEPO-MPs, MOCK-MPs failed to induce hEPO mRNA expression. (PPTX 1940 kb) [file 13287_2020_1932_MOESM1_ESM.pptx]

## Slide 1
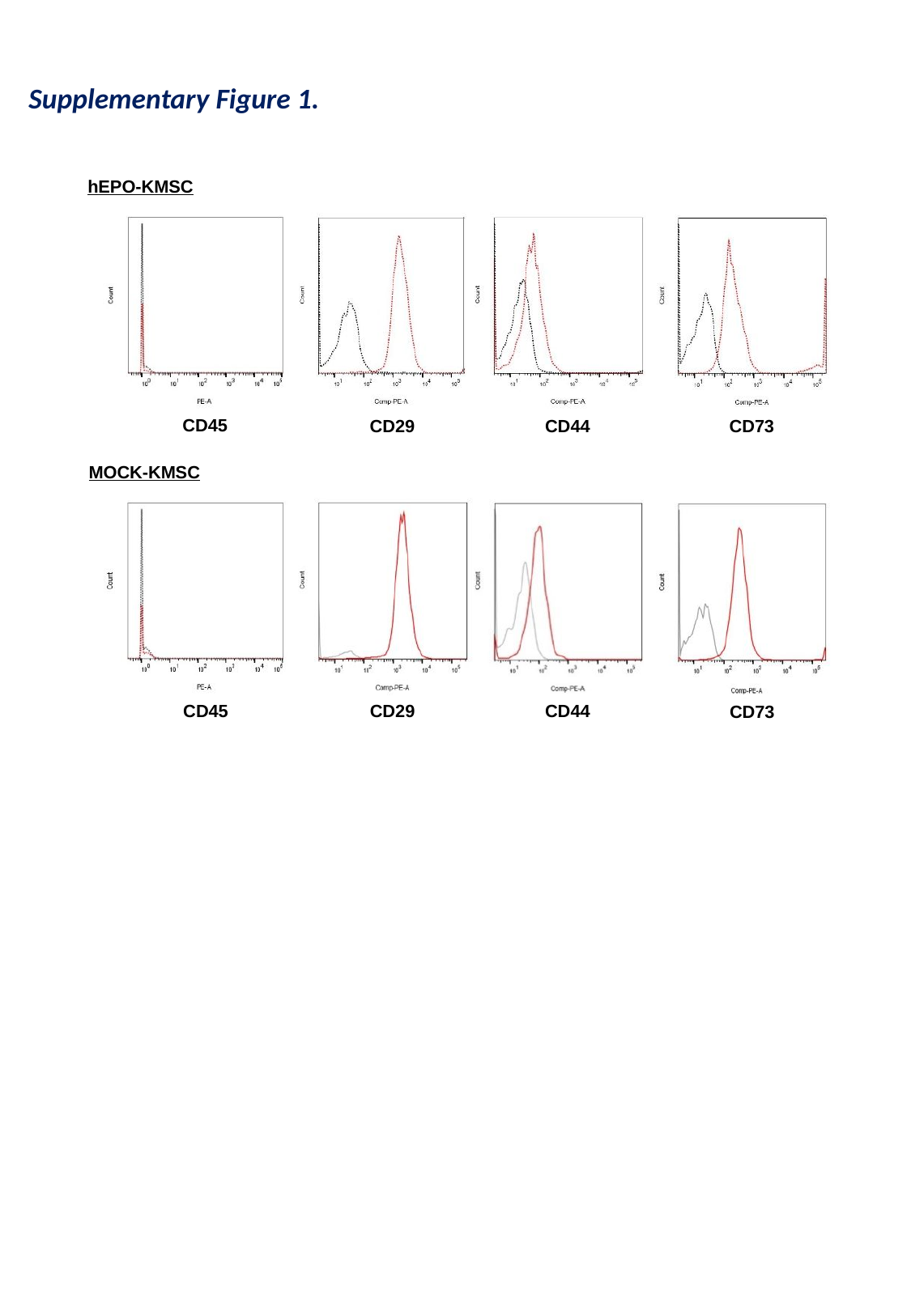

Supplementary Figure 1.
hEPO-KMSC
CD45
CD29
CD44
CD73
MOCK-KMSC
CD45
CD29
CD44
CD73

## Slide 2
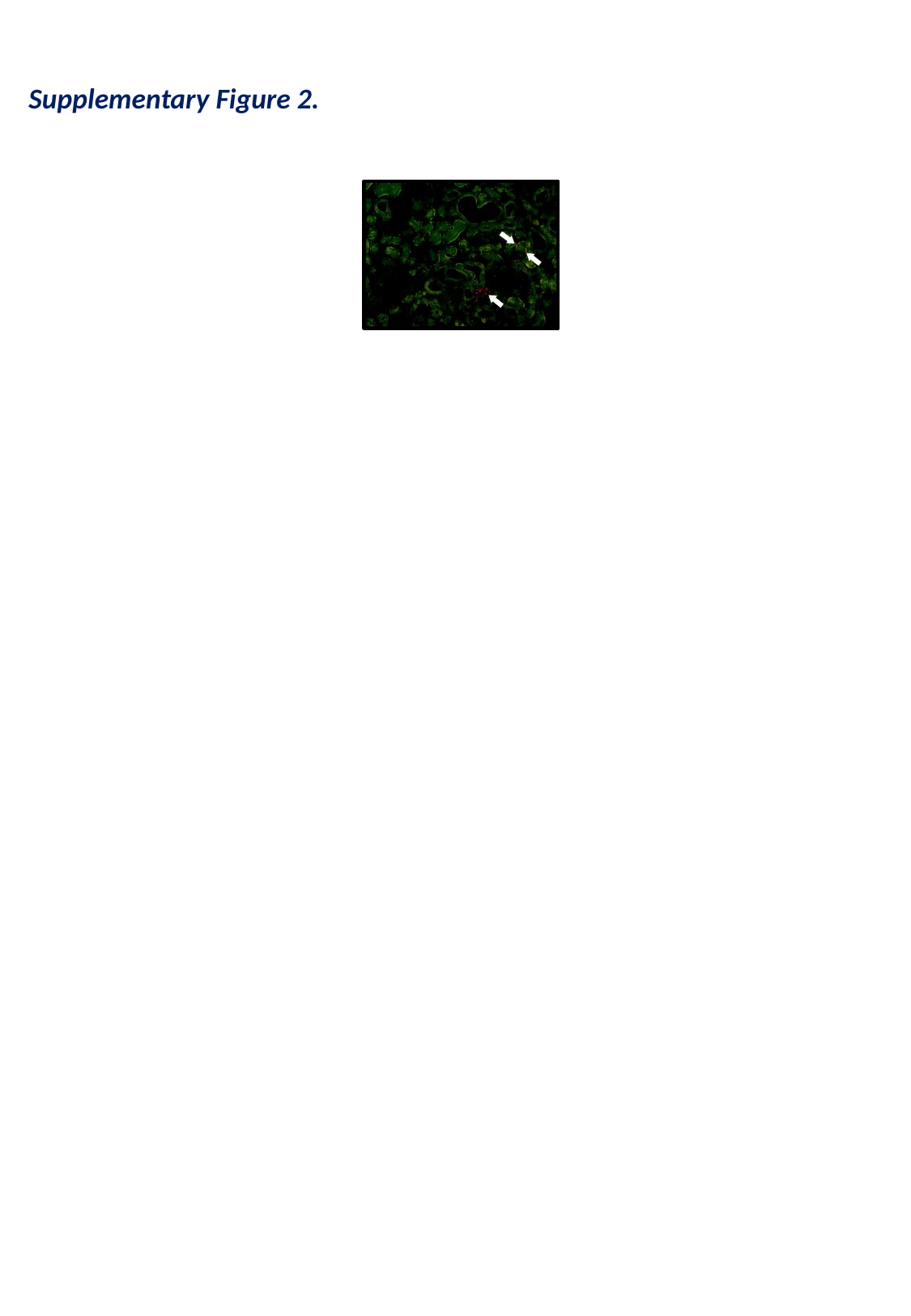

Supplementary Figure 2.

## Slide 3
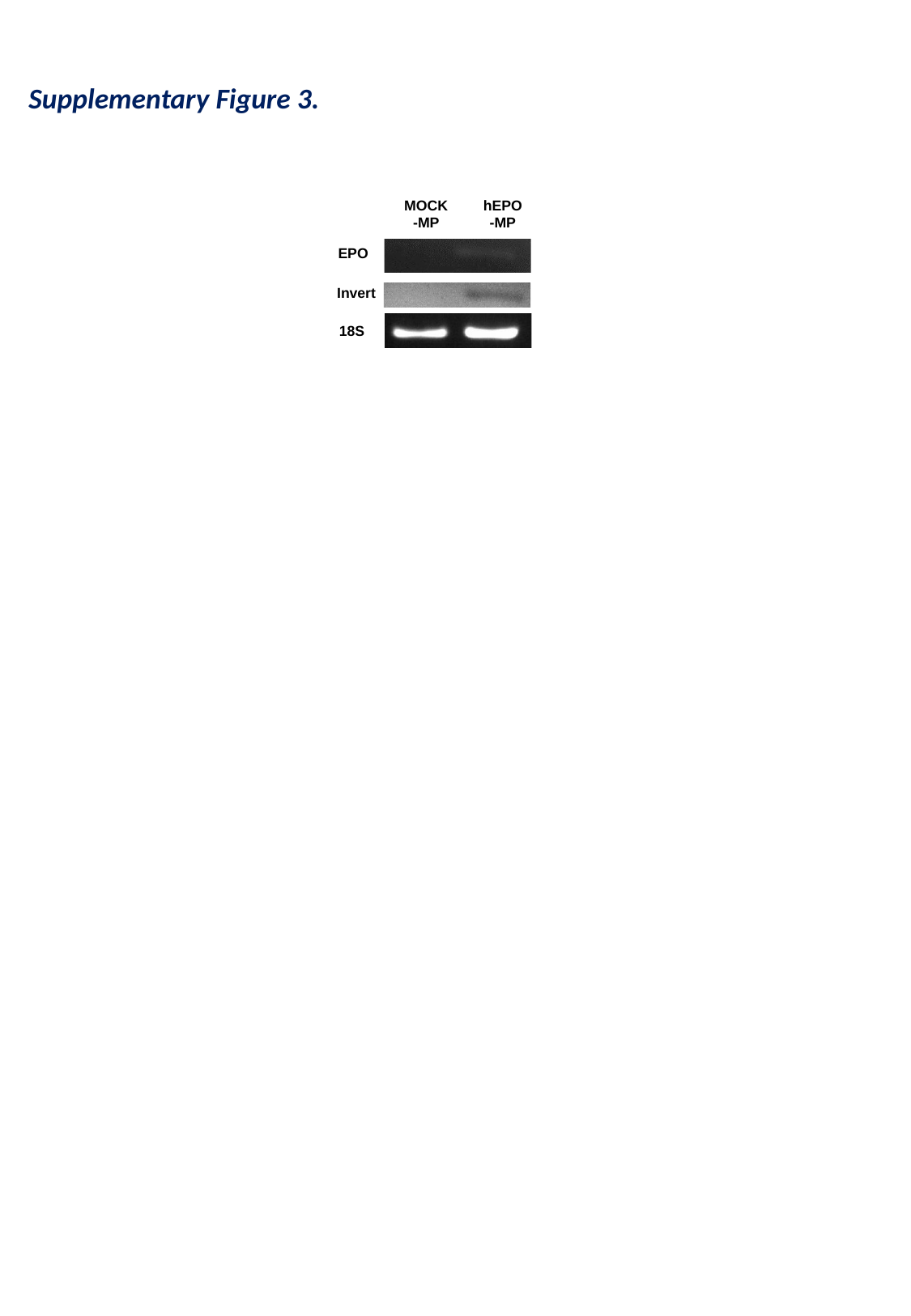

Supplementary Figure 3.
MOCK
-MP
hEPO
-MP
EPO
Invert
18S
